# Understanding relatives’ experience of death rattle

**DOI:** 10.1186/s40359-020-00431-3

**Published:** 2020-06-12

**Authors:** Harriëtte J. van Esch, Martine E. Lokker, Judith Rietjens, Lia van Zuylen, Carin C. D. van der Rijt, Agnes van der Heide

**Affiliations:** 1 Department of Medical Oncology, Erasmus MC Cancer Institute, 3000CA Rotterdam, the Netherlands; 2 Laurens Cadenza, Oosterhagen 239, Rotterdam, 3078 CL the Netherlands; 3 Erasmus MC, Department of Public health, Wytema weg 80, Rotterdam, 3015 CN the Netherlands

## Abstract

**Background:**

Death rattle is a frequently occurring symptom in the last phase of life. The experience of death rattle of relatives has been found to vary. It is unclear if treatment with medication is useful. The most fitting solution for this symptom is still under debate.

**Aim:**

This study aims to better understand the experience of relatives of their loved ones’ death rattle.

**Design:**

A qualitative interview study with a phenomenological approach was performed. Data were collected through semi-structured interviews which were audio recorded, transcribed and analyzed using qualitative content analysis.

**Participants:**

Nineteen family members of 15 patients were interviewed.

**Results:**

Most relatives had experienced death rattle as a distressing symptom. Concerns about how long the rattling would last resulted in more distress. Experience of death rattle was less fierce when other symptoms such as pain or dyspnea prevailed. Hearing the sound of death rattle sometimes reminded relatives of previously witnessed dying trajectories, which seemed to increase their current level of distress. The experience of death rattle is not always influenced by the amount and quality of information given about the symptom.

**Conclusion:**

Death rattle is a stressful symptom and the experience of relatives is influenced by more factors than the sound itself. Communication and information alone seem inefficient to address relatives’ distress. The best approach for dealing with this symptom is unclear. Further research needs to show if prophylactically given drugs may be helpful in its prevention.

## Background

Death rattle is a common symptom in the dying phase which is caused by an accumulation of secretions in the upper airway [1]. This accumulation of fluid (mucus) cannot be easily coughed up or swallowed by the patient, often as a result of a diminished consciousness. The mucus is vibrated by breathing and this creates the typical “gurgling and wet” sound of death rattle [2]. A recently performed systematic review showed that approximately two thirds of dying patients present with this symptom [3]. This review also showed that health care professionals often assume patients are not distressed by the symptom since they are normally unconscious when death rattle develops. However, insight into the impact of death rattle on patients remains unclear and can only be based on the subjective reports of others [3].

Experiences of relatives with death rattle have been studied previously [4–7]. Shimizu found that 66% of the relatives found the symptom “very stressful” and that 55% of them felt a great need to improve care for death rattle. Relatives have been found to associate the sound of death rattle with “drowning”, unworthy dying and to regard the sound “disgusting” [4–6].

Whether relatives experience distress seems to be related to their judgment as to whether a patient is comfortable. On the other hand for some relatives the symptom may be helpful, because it either demonstrates that the patient is still alive, or because it is seen as a sign of impending death and thus the end of the trajectory of suffering [5, 6]. It is not known why relatives experience the symptom death rattle so differently. Nowadays, caregivers usually suggest that the patient is unconscious and therefore probably not suffering from the symptom. However, some authors suggested that this communication may cause relatives feeling themselves not acknowledged in their experiences [8, 9]. A better understanding of the underlying causes of the various experiences may facilitate the support of relatives when death rattle occurs.

The impact of death rattle on the relatives sometimes motivates physicians to start pharmacological treatment for this symptom, even though they assume that the patient is not bothered by it. However, there still is no proven effective medical treatment for death rattle [9–11] and not all health care professionals support the view that drug therapy should be used for this symptom [12].

In order to be able to better support relatives in understanding and dealing with death rattle, it is important to get more insight into their experiences and in factors that influence these experiences. Not only the sound itself, but also the burden of the dying trajectory for relatives and patients, information and communication, and previous experiences may influence how relatives value death rattle.

The aim of this study therefore was to explore relatives’ experiences with death rattle and to gain insight into other factors than the sound alone that may affect this experience.

## Methods

This study is part of a larger study focusing on hydration, death rattle and terminal restlessness in the dying phase. Details of this study, that was performed in 13 clinical sites, are described elsewhere [13]. The study involved four-hourly registration of the intensity of death rattle, for which the scoring scale as proposed by Back et al. was used [2]. These registration data were used to identify relatives of deceased patients in whom the symptom had been present during the last days or hours of life.

### Study design and methodological orientation

We performed a qualitative semi-structured interview study using a phenomenological interpretive approach. Phenomenology is explained by Giorgi as “asking participants from the lifeworld to describe an experience of the phenomenon that the researcher is interested in. What is sought is a description of a situation as lived and understood by the participant from the perspective of the natural attitude“. Phenomenological interpretive approach is a careful reproduction of the participant’s experiences and emphasizes the “how” of this experience [14]. By choosing for the phenomenological approach we aimed to explore the experiences of the relatives based on their own descriptions and terms, rather than by starting from theoretical preconceptions [15].

### Recruitment of respondents

Relatives of patients who had died between 3 and 12 months prior to the moment of recruitment were recruited at the participating study sites. First, the doctors and/or nursing staff were asked to identify patients who, according to the medical record, had presented with death rattle, defined as at least once having had a death rattle score ≥ 1 according to the scoring scale by Back et al. The doctor who had been most involved in caring for this patient was then asked to contact a relative by sending an information letter about the study. If a relative did not respond to the letter within a month, the doctor sent a reminder. Relatives were asked to return a reply card, call the research team or send an email. Relatives who agreed to be interviewed were contacted by telephone by the researcher M.E.L.

### Interviews

All interviews were held by M.E.L. The following topics were explored using an interview guide (see appendix 1):

- Overall evaluation of the dying phase by the relatives
- The experience of death rattle: the sound, the burden for relatives and patients, course and treatment
- Experiences with information and communication about death rattle
- Previous experiences with dying and death rattle

All question in the interview guide were literally asked in every interview. For every topic there were also some prompt questions, which were optional and could be used to help the respondents in their narratives.

### Process

Interviews were held at a time and location of the relative’s choice. Relatives were requested to sign an informed consent form before the start of the interview and for consent to audiotape the interviews. Relatives were informed that all information shared with the researcher was confidential and that all reports would be anonymous. Relatives were offered the opportunity to be referred to their general practitioner or a palliative care counsellor for emotional support following the interview if needed. A few days after each interview the researcher contacted the relative by telephone to evaluate the interview and to assess how they were doing. Interviews were audiotaped and transcribed verbatim and anonymously. Interviews took 45–60 min.

### Analysis

Transcripts were analyzed using a template analysis method [16]. M.E.L. read all interviews after transcription. M.E.L. and H.V.E. coded the first interview independently. Next, they constructed a template (version 0) with themes based on these initial codes. H.V.E. reread the first interview and recoded it based on this template, which resulted in reformulation of several themes. M.E.L. and H.V.E. discussed the changes in the template and agreed on the next version of the template (version 1). H.V.E. started with coding the other interviews according to this template. During this coding process, two new themes emerged in the following three interviews which were discussed by M.E.L and H.V.E. and added to the template (version 2). These themes appeared to occur in almost all of the following interviews. Therefore, it showed these themes were common for the respondents. The first interview was read again by H.V.E., without occurrence of the two themes. The final template consisted of 13 themes. All these themes were described in detail for analyzing. All authors agreed on this template. (see appendix 2).

### Ethical approval

This study was approved by the Medical Research Ethics committee of the Erasmus MC, University Medical Center Rotterdam, the Netherlands (MEC 2013–364).

## Results

We approached relatives of 95 patients. Of those, 19 relatives of 15 patients were willing to be interviewed. Relatives of 30 patients refused and those of another 50 patients did not respond to the information letter.

Nineteen family members of 15 patients were interviewed between February and June 2014. All relatives choose to be interviewed at home. We started the interview by asking for the experience of the relative of the disease trajectory of their loved one. Their narratives included comments about communication, information, quality of care by the healthcare professionals, symptoms and the dying phase. We then asked more in depth about the different experiences by literally following our interview guide. When the dying phase was discussed, we immersed ourselves in the symptom death rattle. Interviews took 45–60 min. During the interview, the relatives also revived the last phase of their loved one, often accompanied by melancholic emotions. Support was offered after the interviews.

The characteristics of the relatives who were interviewed and the patients are shown in Table 1.

**Table 1:** Characteristics interviewed relatives and patients

Relative	Relation to the patient	Patient’s place of Death	Patient’s age (range)	Time between Interview and patient’s death (months)
1	Niece	Hospice	80–89	9
2	Daughter	Hospice	80–89	9
3	Daughter	Hospice	80–89	9
4	Husband	Hospital	50–59	7
5	Daughter	Hospice	60–69	7
6	Husband	Hospice	60–69	6
7	Wife	Hospital	60–69	10
8	Son			
9	Husband	Hospital	50–59	12
10	Wife	Hospital	70–79	9
11	Daughter			
12	Wife	Hospital	60–69	12
13	Son			
14	Wife	Hospice	80–89	7
15	Daughter	Hospital	70–79	8
16	Wife	Hospital	60–69	12
17	Wife	Hospital	60–69	12
18	Husband	Hospital	80–89	8
19	Daughter			

Nine patients had died in the hospital whereas six had died in an inpatient hospice. Relatives were patients’ spouse [10], child [8] and one niece. They were interviewed on average 9 months after the death of the patient. Most patients had been diagnosed with cancer. Their age at death varied from 55 to 89.

Most themes followed from the topics of the interview guide: the experience of death rattle, previous experiences of death rattle, information about the symptom death rattle and the burden for the patient. The two themes identified while analyzing the interviews were: concerns about how long the rattling would last and the impact of other symptoms.

### The experience of death rattle

The experience of death rattle involved three separate elements: the sound of death rattle, concerns about choking, and the association with dying.

All relatives described their experience of the sound of death rattle in neutral or negative terms.

“I found it irritating, not annoying.” (*participant 14*)

“And this rattling, it didn’t bother me, a little mumbling ….” *(participant 4)*

Other relatives used more negative terms.

“I found the rattling confronting, because it is a distasteful sound, no matter how you look at it” *(participant 13)*

Some relatives used emotional wording to describe their experience:

“I watch her lying in bed, almost upright and completely cramped, resting at her pillow, with an open mouth, gasping for breath, making a rattling sound. It was so intense, I got down on my knees and thought ‘ if I do not watch out now, I will break down emotionally’” *(participant 6)*

Other words used to describe the experience were “horrific”, “inhuman”, “awful” and “unpleasant”.

A number of patients associated the sound of death rattle with “choking” and were concerned that the patient was suffocating.

“I asked, because my father was rather short of breath: he will not suffocate will he? That was my fear.” *(participant 2)*

Death rattle was recognized as a sign of approaching death by several participants.

“… … Horrible, … it was the first time for me to see someone die and to be there from the beginning. But this *(the sound)* is something you associate with dying. So that sound, yes it will stay with me forever. *(participant 14)*

### Previous experiences with death rattle

Several relatives had previously witnessed the death of a relative who had presented with death rattle. They recognized the sound and knew that rattling means that dying is nearby. They described that experience.

“Actually I have experienced that *(death rattle)* with a brother-in-law … .. whom I loved very much ..... And I still see him .. he was sitting on a pile of cushions, rattling, and I thought ‘he won’t be living long anymore’ …. I still remember this very well … ... So when my husband started rattling, I thought ‘yes .. there it is again ....’, ... and then I thought ‘stop …. it is as if my brother-in-law is dying again’. *(participant 17)*

“… this was not the first time, I have experienced this (rattling) before. And every time I think ‘Good lord, what is this, why does this have to be this way? Why do we do this to people?’” *(participant 5)*

### Information/communication

All participants had received information about the dying phase and associated symptoms, especially death rattle. Some had also received a booklet with information about the dying phase and its symptoms. Health care professionals had used normalizing phrases (‘death rattle is a part of dying, patients are not bothered by it’). We found that the experience of death rattle did not seem to be influenced by the amount and quality of information given by healthcare professionals.

*Participant 6*

“Nurses had told me that she may present with death rattle. And that I did not have to worry about it ….”

“Don’t worry about it, come and look at her … she is not aware of it, she is far away, believe me, she is not aware of it.”

“I watched her lying in bed, almost upright and completely cramped, resting at her pillow, with an open mouth, gasping for breath, making a rattling sound. It was so intense, I got down on my knees and thought ‘if I did not watch out now, I will break down emotionally’”.

*Participant 12 and 13*

“…… … A lady came with a computer and explained the symptoms and the process (of dying) … … it was all in the leaflet … and he literally died like it was described”.

“.. And it was confirmed by the nurses on the ward, who said things like: it is what it is, we can’t do anything, it is a part of dying. He is not suffering.”

“It was horrible, horrible … …”

### Burden for the patient

For most participants, it was not clear whether patients suffered from the death rattle. Some participants stated to have observed their loved one and had seen no signs of stress. They then seemed to be better able to handle the death rattle.

“I don’t think she was bothered by it. She was lying there, very calm.” *(participant 14)*

Other participants were uncertain about the suffering of their loved ones, despite the fact that there were no signs of distress.

“… Because I would like to know, the person who is lying there, how does he experience it. Because I may think that it is terrible, but maybe the one who is lying there is not bothered at all. Or does not experience it that way. But we will never know. It is only what we see and our own interpretation of it, that is how I see it … ..” *(participant 5)*

“… The noise of this rattling was very annoying …. , every moment you think will he suffocate, or does it bother him? Yes, you obviously do not know that … .and you cannot ask him ….” *(participant 15)*

### Concerns about how long it would last

Relatives were concerned about how long it would take for the patient to die. This uncertainty seemed to make the experience of the sound of death rattle more pronounced.

“He rattled until he died, from half past three until half past six, it has not been long. You just wait and wait until it (the rattling) stops … .. I didn’t like it. And I was just waiting, hoping it wouldn’t take too long.” *(participant 17; death rattle score 1, 2 measurements in time)*

“…. it was really a drama .... because that Thursday that I went there, I came in and he was already rattling and I literally thought: this is the beginning of the end … I cannot take this sound another 24 hours and I thought: I’m not going to sit here and listen and watch him until it’s over … … because I find this horrible to watch ….” *(participant 5; death rattle score 2, 3 measurements)*

“… and then this rattle, this gurgling … ..you hear him breathe, a long pause and then ‘grrgrr’ (*mimicked the sound),* for two days in a row … .., while everybody is saying that when you hear this rattle death is near … ..Two days then is a long time ….” *(participant 13; death rattle score 3, 11 measurements)*

### Impact of other symptoms

The experience of death rattle seems to be less prominent when other symptoms prevail. Participants felt more burdened by the patient suffering from other symptoms than by death rattle. Situations were described in which pain, dyspnea or restlessness were more stressful than death rattle.

“He was dying, we knew that. And if the rattle is a part of dying, then so be it … at that point you are just grateful that with some extra morphine he can sleep peacefully and no longer feels pain … well than I can handle a little bit of rattling.” *(participant 11)*

“The rattling itself is basically no problem, because a patient goes to sleep and snores or mutters or whatever, but when you see from the body that it bothers her, that she is restless, , that she has pain, is uncomfortable, short of breath, rattling … … … She did not recognize me anymore, she did not open her eyes either … … it was terrible.” *(participant 1)*

## Discussion

We found that death rattle can be a stressful symptom for relatives, which confirms findings from previous studies [4–6]. We found no positive experiences of the sound of death rattle, in contrast to others [4]. The experience of relatives who valued death rattle as burdensome is affected by more factors than the sound alone. Previous experiences with death rattle, insecurity about the burden for the patient and a longer duration of the death rattle seem to negatively influence relatives’ experiences. Relatives who did not value death rattle as burdensome did not describe these experiences. All participants felt that informing them about death rattle had a limited impact on their experience of the symptom. Symptoms such as pain, dyspnea and delirium seem to reduce the impact of the experience of death rattle.

### Relationship to prior research

Death rattle is a symptom that has been suggested to be more bothersome for relatives than for the patient. It is an experience in which all senses are stimulated: relatives can hear the rattling, they can see the mucus drip and they sometimes can smell this mucus. The sound and look may result in a feeling of distance in a period in which intimacy is important. Campbell (2018) suggested that ‘normalization’ of the sound of death rattle is better than medical treatment to assuage family members’ and clinicians’ distress [12]. Wee and Hillier [5] and Shimizu [4] found that frequent explanations and understanding relatives’ interpretations of the sound is important to alleviate the burden of death rattle. However, our results indicate that the experience of death rattle may not be influenced by information given to the relatives. Normalizing the sound does not seem to sufficiently alleviate the experience of relatives in all cases either. In this study, we see that other symptoms sometimes have more impact on relatives than death rattle.

### Strengths and limitations

A strength of this study is that it is the first attempt to identify factors which influence the experience of death rattle in addition to the sound alone. By interviewing all relatives at home, we created an atmosphere in which they felt comfortable and free to share their experiences. The impact of death rattle for loved ones became very clear. Their vividly described experiences suggest that, although time had passed, the experience of death rattle was still very much alive.

Our study has some limitations. First, the response rate was low, which may have been caused by practical reasons as a rehousing of the relatives, mail getting lost or ignorance of relatives for an information letter. Second, self-selection may have been a problem. Relatives may not have wanted to participate and share their experiences, because they experienced a stressful dying process with many burdening symptoms and suffering.

And finally, the timing of the interviews varied from 3 to 12 months after death. The months between death and the interview can alter bereaved relatives’ memories and feelings about the dying process [17, 18].

### Implications for practice and future research

Not all relatives valued death rattle as burdensome, but for those who did, the experiences were influenced by more than just the noise. Further research on how to manage death rattle is needed, because of the burden for some relatives and uncertainty about the burden for the patient. Drug treatment could be considered to manage death rattle. Previous research has suggested that anticholinergics can be used to diminish death rattle, but variable effects have been shown [9–11]. In these studies the medication was started when death rattle arose. It has been suggested that these drugs could be more effective when they are administered prophylactically, that is, before death rattle occurs [11, 19]. One recent, non-placebo controlled study found that prophylactic anticholinergics for death rattle may be effective [19]. A placebo-controlled RCT on prophylactic use of scopolaminebutyl is currently being conducted [20].

At this moment, communication and information about death rattle is what health care professionals can offer the relatives. The phenomenon of death rattle should be addressed by health care professionals by acknowledgement of former experience of patients and relatives, and by providing accurate information and communication. Information should consist of a clear explanation of the symptom and the probably limited degree to which patients suffer from it, confirmation that the patient is in the dying phase, and an explanation of the measures that can be taken to diminish the sound. This information must be repeated regularly (if necessary more than daily).

## Conclusion

Death rattle can be a stressful symptom for relatives that is influenced by more factors than the intensity of the sound alone. Adequate information and communication cannot always relieve the burden for relatives. Further research is needed to show if prophylactically given drugs may be helpful in preventing this stressful symptom.

## Data Availability

The datasets used and/or analyzed during this study will be available from the corresponding institute on reasonable request.

## Appendix 1. The Interview guide

### Final months of the patient

1. Can you tell me about the last months of your relative?
  - Prompts:
    - Was your relative sick?
    - Has he / she been treated for this disease?
    - Did your relative need personal care during the last months?
    - Who was involved in that care (you, professionals, others)?
    - Where did your relative stay (home, institution) and did this change in the last months?

### Evaluation of the course of the dying phase

2. How do you look back on the death of your relative?
  - Prompts:
    - What are the reasons that you consider it a good/bad death?
    - What went well, what went less well, what could have been better?
3. How were you involved in the care of your relative?
  - Prompts:
    - Did you do personal care of your relative?
    - Did you wake?
    - Other ways?

### Course of the dying phase / Information and communication

4. Can you tell me about the course of the last days (the dying phase) of your relative?
  - Was there a moment at which the doctor/health care professional told you that your relative was dying?
5. Did healthcare providers explain what you could expect?

*If so, was it told at the start of the dying phase that death rattle could occur? What did you think of that?*

- Prompts
  - What kind of **symptoms** and problems did your relative experience in the last days?
  - Did your relative have a rattling noise when breathing? / **Did** your relative **rattle**?
  - How **has** the death rattle **been dealt with**?
    - o What did the caregivers told you about this symptom?
    - o Was treatment started to treat this death rattle?
- What was the course of this symptom during the dying phase?

(extensive, continuously present, varying in severity)

- How was it for you to hear your relative make this noise? (negative - neutral - positive)
  - o What **did you think** when you heard him/her rattle?
  - o What **did you feel** when you heard him/her rattle?
- Do you think **your relative** suffered from this symptom?
  - o Can you explain why you think that?
- Do you think that other **bystanders (loved ones / patients)** suffered from this symptom?
  - o Can you explain why you think that?
- Do you think that **the caregivers (doctor / nursing staff)** suffered from this symptom?
  - o Can you explain why you think that?
- **How do you look back** on the experience of death rattle? (same, different)

### Previous experiences

6. Do you have other experiences with a loved one dying?
  - Prompts:
    - How did that go?
    - Did this person have a rattling noise when breathing? / Did this person rattle?
    - What was it like for you to hear this person make this noise? (feelings / thoughts)
    - How do you look back on that now? (same, different)

### Finally

7. How are you now?
  - Prompts:
    - Mourning?
    - Physical and mental functioning?
    - Did you receive support after the death of your loved one?

## Appendix 2. Template Diagram

- Theme 1. The tipping point to death *(descriptions of deterioration resulting in hospice admission or clear marking of the last phase)*
- Theme 2. Information *(communication/information and actual conversations about the following)*
  - Recognizing the last phase *(unconscious / incapable of recognizing deterioration / awareness and recognizing deterioration)*
  - The dying phase *(sleepy or diminished consciousness, drinking sips, difficulty with swallowing: signs of the dying phase)*
  - Marking the dying phase *(indicate clearly that the dying phase has arrived)*
  - About rattle *(explanation of the symptom, chance for occurrence)*
- Theme 3. Description of the process of dying *(“like a candle”, with clarity, dying fast/unexpected/ degrading)*
- Theme 4. Acceptance of death *(what were the respondent’s feelings about this approaching end: relief, fear, denial, etc)*
- Theme 5. Symptoms in the dying phase *(pain, dehydration, unconsciousness, pressure sores, death rattle)*
- Theme 6. Presence of respondents in the last days *(continuously, unregularly)*
- Theme 7. Experience of respondents *(how was the experience with …)*
  - With dying *(fear of death rattle, fear of dying itself, violent to wait for death, fear with other breathing,* etc.*)*
  - With death rattle *(unsavoury, confrontational, embarrassing, nasty, fear, no burden)*
  - With death rattle over time *(retrospective, what is death rattle meaning now?)*
  - Death rattle influenced/coloured/marked by external factors *(previous experiences)*
  - With the care provided/given (*what was done and how was it experienced)*
- Theme 8. Death rattle burden for patient through the eyes of the respondent *(probably not bothered by it, unknown, difficult to say, guessing, yes/no)*
- Theme 9. Experience of others with death rattling through the eyes of the respondent
  - Family/friends *(how were they experiencing according to the participant?)*
  - Healthcare professionals *(how were they experiencing according to the participant?)*
- Theme 10. Death rattle treatment *(medication, change of posture,* etc.*)*
- Theme 11. Death rattle *(now and then, continuous, changes in sound*)
- Theme 12. Wishes around end of life and dying of patients
- Theme 13. Mourning *(how are respondents feeling at the moment of interview)*
